# Meditation as an Adjunct to the Management of Multiple Sclerosis

**DOI:** 10.1155/2014/704691

**Published:** 2014-07-01

**Authors:** Adam B. Levin, Emily J. Hadgkiss, Tracey J. Weiland, George A. Jelinek

**Affiliations:** ^1^Department of Medicine, The University of Melbourne, St Vincent's Hospital, Melbourne, VIC 3065, Australia; ^2^Emergency Practice Innovation Centre, St Vincent's Hospital, Melbourne, VIC 3065, Australia; ^3^Department of Epidemiology and Preventive Medicine, Monash University, Melbourne, VIC 3004, Australia

## Abstract

*Background*. Multiple sclerosis (MS) disease course is known to be adversely affected by several factors including stress. A proposed mechanism for decreasing stress and therefore decreasing MS morbidity and improving quality of life is meditation. This review aims to critically analyse the current literature regarding meditation and MS. *Methods*. Four major databases were used to search for English language papers published before March 2014 with the terms MS, multiple sclerosis, meditation, and mindfulness. *Results*. 12 pieces of primary literature fitting the selection criteria were selected: two were randomised controlled studies, four were cohort studies, and six were surveys. The current literature varies in quality; however common positive effects of meditation include improved quality of life (QOL) and improved coping skills. *Conclusion*. All studies suggest possible benefit to the use of meditation as an adjunct to the management of multiple sclerosis. Additional rigorous clinical trials are required to validate the existing findings and determine if meditation has an impact on disease course over time.

## 1. Introduction

Multiple sclerosis (MS) is one of the most common chronic neurological diseases, affecting between 1 in 500 to 1 in 1500 people in Australia, Europe, and America [1, 2]. The condition is characterised by neuronal demyelination and inflammation, leading to axonal injury as the disease progresses [2, 3]. MS has several forms, the most common (70–80%) being relapsing-remitting. Other forms include primary progressive, secondary progressive, relapsing progressive, and benign [4]. MS has a typical onset between 20 and 40 years of age, having a significant impact on quality of life (QOL) over the duration of the disease. The disease manifests with a range of physical and neurological symptoms [5] including sensory loss, optic neuritis, motor weakness, diplopia, and limb ataxia [6], as well as an increased risk of stress, anxiety, and depression [7]. MS is considered incurable; however, many strategies exist to help manage symptoms and alter the disease course including pharmacological agents, lifestyle risk factor modification, rehabilitation, and psychosocial support all of which have varying efficacy [8].

Although meditation in all its forms has been around for centuries, interest in the practice as a medical treatment in the Western world has been more recent. The interest was particularly sparked by Kabat-Zinn's research into mindfulness meditation suggesting that meditation could be a useful tool for treating chronic pain [9].

Meditation is a term that encompasses a wide range of techniques such as mindfulness-based meditation (including mindfulness-based stress reduction (MBSR) and mindfulness-based cognitive therapy (MBCT)), mantra meditation (including transcendental and clinically standardised meditation), and many more [10, 11].

In its various forms, meditation has been shown to be associated with symptom reduction in medical and psychiatric conditions, electroencephalography (EEG) changes, and beneficial structural brain changes on neuroimaging with long term use [11–17]. While meditation has been shown to moderate stress, anxiety, and depression, studies have often been poorly designed, with biased patient selection, poor controls, and small sample sizes among other deficiencies detracting from the credibility of the literature [18]. MS is associated with significant rates of depression [7, 19–21], anxiety [22], stress, chronic pain [23, 24], and fatigue [25] which are often important predictors of QOL [7, 26]. Stress and other mental health-related comorbidities appear to play a role in increasing relapse rates and decreasing QOL in MS [27, 28].

Yet, few studies have examined the effect of meditation on mental health outcomes or QOL in MS, despite its great potential benefit.

This integrative review aims to examine the existing peer-reviewed literature on the use of meditation in MS to reduce depression, anxiety, stress, chronic pain, and fatigue and whether meditation can lead to improved QOL for those with MS.

It also aims to gather evidence on the effectiveness of meditation as secondary prevention for MS morbidity. Finally, it aims to identify gaps and limitations in the current literature.

## 2. Methods

This review of literature published prior to March 2014 was undertaken using PubMed, PsycINFO, the Cochrane library, and Google Scholar. Search terms used were multiple sclerosis, MS, meditation, and mindfulness (all were searched as MeSH terms). The Boolean operator term OR was used to search [mindfulness OR meditation]; these results were then combined with the Boolean operator term AND to search [(mindfulness OR meditation) AND (multiple sclerosis OR MS)].

Articles which cited a key randomised controlled trial by Grossman et al. [29] were searched on Google Scholar. Only English language articles were included; no other limits were set.

Primary literature was selected only if the patient sample had MS and the assessment of mindfulness or another form of meditation (hypnoses and complementary and alternative therapies were excluded) was examined in the study, including both observation and intervention studies. Intervention studies were accepted regardless of whether or not there was a control group. In the context of an integrative review, all qualitative and quantitative research was selected (assuming it met the criteria above) regardless of whether validated tools were used to measure outcomes or not. In studies where MS and another medical condition were examined, measures specific to the MS group were reported, where possible.

The reference list of all studies that were accepted for the review was analysed to see if there were any additional relevant papers that had not been found using the search methods above.

All search results were reviewed by a single reviewer (AL) to determine relevance to the criteria; included papers were then reviewed by a second reviewer (EH) and any discrepancies were resolved by a third reviewer (TW). The assessment of intervention and observation studies was guided by the PRISMA guidelines [30] and the STROBE guidelines [31], respectively. Selected papers were summarised in Table 1, highlighting study type, study focus, methodology, participant information, control details (if applicable), outcomes, and limitations.

## 3. Results

Using the search criteria 19 results were found on PubMed, PsycINFO, and the Cochrane library and 9030 results were found on Google Scholar. The first seven pages of Google Scholar articles were scanned to find relevant articles. There were also 72 articles which cited Grossman et al. [29] on Google Scholar.

Using the selection criteria 12 pieces of primary literature were selected to be included in the literature review. Of these, two were randomised controlled trials [29, 32], four were cohort or pre- and postinterventional studies [33–36], and six were surveys [37–42] (Table 1).

### 3.1. Randomised Controlled Trials

Grossman et al. conducted a randomised controlled trial of 150 patients residing in Switzerland with relapsing-remitting or secondary progressive MS. Cases were assigned: one 2.5 hour session of mindfulness meditation a week for eight weeks, a one-day seven-hour session, and 40 minutes of meditation homework daily. Controls continued their usual care. High attendance rates and low dropout rates were reported for the intervention group. It was found that, using validated self-report measures, the meditation group had significant improvements in QOL and significantly lower rates of fatigue (MFIS), depression (CES-D), and anxiety (STAI) when compared to the control group [29] after intervention and at a six-month follow-up. This trial was soundly constructed and provides reliable evidence that meditation may have a beneficial effect on QOL and mental health-related comorbidities [43]. The major limitation of the study was the lack of a sham control group, which meant that the “self-efficacy effect” may have influenced the results [44].

Mills and Allen conducted a randomised controlled trial on patients with secondary progressive MS, with 12 participants randomised to the meditation group and 12 to the control group. The meditation group received an hour of one-on-one meditation sessions for six weeks as well as handouts, tapes, and written material. The control group continued on normal therapy. At the completion of the mindfulness course and at three months after intervention, self-reported MS symptoms were found to have significantly improved in the intervention group. Each participant also had a relative or a friend assess their symptoms before and after intervention and significant improvements were also noted with that measure. Given the small size of the study and a relatively large dropout rate, the results must be treated with caution. Additional limitations include not having any sham control and follow-up being limited to three months [32].

### 3.2. Pre- and Postintervention Studies

In a longitudinal study, Hadgkiss et al. and Li et al. found that a five-day live-in course which focussed on lifestyle modifications including diet, sunlight exposure, exercise, and meditation improved physical and mental HRQOL compared to baseline; effects were followed and found to be significant at one, two and a half, and five years after the course was completed [33, 34]. While it was conducted thoroughly, the effect of, and adherence to, meditation specifically was not differentiated from the other program recommendations. The study would also benefit from being repeated with a control group.

Tavee et al. performed an intervention study of meditation in MS patients, with the intervention group undertaking weekly meditation classes for two months and control subjects receiving standard care. It was found that those who participated in meditation had significantly less pain, improvements in cognitive and psychosocial aspects of fatigue, and improved QOL after intervention but no statistically significant change in mobility. The study was limited by a small sample size (less than 20 patients with MS), lack of long term follow-up, and lack of a sham control group. Furthermore, it was not randomised meaning that those who chose to be in the meditation group may have had an increased perception of the beneficial effects of meditation [35].

Pritchard et al. conducted a study which demonstrated decreased stress compared to baseline after a six-week meditation course. Stress was assessed on a validated scale and shown to significantly decrease after the course, suggesting an association between meditation and decreased stress levels in MS [36]. The study was limited by a small sample size (only 12 MS patients), no control group, and lack of long term follow-up.

### 3.3. Surveys

In a survey of over 3000 MS patients conducted by Nayak et al., it was found that 12% of the survey responders had tried meditation, with modest efficacy (determined by a self-reported five-point scale of reduction in perceived disease severity) [37]. Participants reported pain, overall symptoms, and fatigue as the top three issues that meditation was helpful for. This is one of the largest studies of its type and probably represents the best estimate of meditation use in MS in the Western world. Berkman et al. found that 22.9% of survey responders had tried meditation, reporting benefits of stress relief, relaxation, lower fatigue, strengthened immune system, and, interestingly, slower disease progression [38].

In an online survey of 2529 patients with MS, which looked at a range of issues relating to the disease, Simmons et al. found that 9% of respondents had ever practised meditation and reported that it improved their MS. Interestingly, a small number of respondents (0.2%) reported that meditation worsened their MS symptoms [40].

In another large online survey Skovgaard et al. also found that 5% of responders had used meditation in the previous 12 months to help treat their MS symptoms. The survey found that respondents who had tried any form of complementary or alternative medicine were most likely to be females aged between 18 and 40 years old, which was not surprising given this is a significant proportion of the MS demographic. It was also found that meditators were more likely to have been university-educated and have a higher income [39].

Senders et al. undertook a cross-sectional survey examining trait mindfulness and its effect on various outcomes, all assessed using validated tools. They found that having a mindful disposition (as assessed by the Five Facet Mindfulness Questionnaire (FFMQ)) was significantly associated with decreased perceived stress (model accounted for 25% variance), increased resilience (44%), increased adaptive coping (11%), decreased maladaptive coping (29%), and higher mental health QOL (20%). It should be noted that participants FFMQ scores were independent of whether they had participated in formal meditation practice or not, and as such the variation in the above mentioned scores cannot be solely attributed to participation in meditation [42].

In a mixed methodology study, incorporating a survey of attendees at an MS congress and focus group of attendees at a “complementary therapies and MS” workshop, conducted by Esmonde and Long, 25% were found to have participated in relaxation and meditation activities in the previous 12 months. Reported benefits included improved sleep, reduced spasticity, easing of muscle tension, and increased sense of well-being. Relaxation and meditation were reported to be at least somewhat helpful in relieving MS symptoms, with over a quarter rating it as extremely helpful. Participants in the workshop may have been more likely to ascribe benefits to the practice of relaxation and meditation as they may have been more open to trying nonpharmacological therapies. The study was also limited by the use of a nonvalidated survey and having participants discuss their experiences in small groups rather than individually, which may have led to unease discussing personal experiences, especially if they were negative. Nevertheless, the reports of benefits for some patients are an indication that meditation may be a useful augmentative treatment in MS [41].

All the above mentioned surveys had limitations. A common limitation was that of participant selection. Surveys were sent to large cohorts and were voluntarily-completed leading to a selection bias as patients who had tried meditation and found it effective may have been more likely to commence and complete the surveys. Furthermore, results were limited by the self-reporting nature of surveys, which could have been biased by poor recall or under- or overestimation of responses. Some useful analyses within these papers did not distinguish meditation from CAM therapies, thus limiting the interpretation of findings.

In some surveys it was difficult or even impossible (in the case of online surveys) to verify the MS diagnosis [37, 39, 40]. Additionally, many of the survey populations were limited to one or two countries [37, 39, 41, 42], meaning that the findings may not be generalisable to the larger population of patients with MS.

Some of the studies did not employ validated instruments, so the reliability and validity of results are not clear [37, 40, 41]. Additionally, some surveys had a small sample size [41, 42], which limited reliability and statistical precision of the findings.

Finally, all of the surveys were cross-sectional from which causation or change over time cannot be deduced.

## 4. Discussion

The literature shows that stress plays a significant role in MS pathogenesis and progression. Past studies have found that 85% of MS exacerbations were associated with stressful life events, strongly suggesting a link between stress and disease course [46]. Further, multiple meta-analyses and systematic reviews have found that while there are other factors that contribute to morbidity, such as viral infections, stress plays an important role in the number of relapses patients with MS experience and is often underrecognised [27, 47, 48]. Mohr et al. demonstrated that stressful life events were associated with new brain lesion formation (assessed with MRI) four to eight weeks after a moderate stressor [49].

Cortisol, which is released by the hypothalamic pituitary axis in response to stress, is often chronically elevated in MS patients. As a result of chronically increased levels of cortisol, it has been proposed that MS patients form resistance to the hormone and as such do not benefit from the normal anti-inflammatory effects of the hormone, which would otherwise help to allay their symptoms [50].

Meditation has been shown to decrease cortisol levels and improve sleep for both novice and experienced meditators. Given that cortisol plays a key role in stress modulation, the results suggest that the beneficial effects of meditation may operate partly through this mechanism [51]. Additionally, meditation has been shown to be associated with activation of the prefrontal cortex and the anterior cingulated cortex, which are areas of the brain associated with concentration as well as altering EEG tracings in many medical conditions [14]. These changes are associated with decreased levels of stress.

Given that stress has been shown to be implicated in relapses in MS [46, 49] and that meditation has been shown to relieve biological markers of stress [14, 51] it is reasonable to hypothesise that meditation may have a direct impact on MS disease course through its effect of modulating the stress response, although this has not yet been studied in detail.

Because stress and depression affect QOL in people with MS and may lead to disease exacerbation [46], there has been interest in methods of reducing stress in MS [52], including meditation. There have been systematic reviews on the effects of meditation as well as other “mind-body interventions” on disease course in MS as well as other neurological conditions [53–55], including a recent systematic review looking at the use of meditation in MS specifically [56]. This latter systematic review however considered only randomised controlled trials; given the paucity of these in the literature, the review may therefore be considered to have too narrow a focus. Integrative reviews in contrast take a broader view including qualitative and quantitative research as well as discussion papers and grey literature [57]. Given the difficulties in conducting randomised controlled trials in this area, the broader approach we have adopted is necessary to better synthesise current knowledge and help develop a more realistic view of the effects of meditation on MS. Integrative reviews on meditation as a therapeutic technique in MS have been lacking, thereby warranting the present review.

Our review shows that a substantial proportion of people with MS either have tried meditation or meditate regularly. The available body of research has consistently found meditation to be of benefit to those with MS, with very few or no harmful side effects.

Despite weaknesses in the evidence base, meditation appears to have important benefits in improving QOL as well as potential benefits for the management of pain, stress, fatigue, and depression risk in people with MS. There is currently however limited evidence to suggest that meditation has an effect on relapse rates or other markers of disease course, although it is biologically plausible and this may be shown to be true in future better-designed studies.

*Limitations of the Review*. Given that meditation is an umbrella term for many different practices, there was a degree of heterogeneity between studies making it difficult to directly compare between different cohorts. Many studies had limitations, most notably self-reported outcomes, failure to use well-validated tools, and, in the case of randomised controlled trials, the absence of sham intervention. The literature review was further limited by a small number of primary resources directly relating to meditation and multiple sclerosis. Finally, only English language literature was included.

Despite these limitations, overall the evidence supports a beneficial effect of meditation on symptom management and QOL among people with MS. It seems that meditation may have an important role to play in the integrated management of MS. Further studies on meditation in MS are required, preferably randomised controlled trials utilising some sham form of therapy.

## 5. Conclusions

MS is a disease which can have significant negative effects on QOL. MS is associated with a higher risk of depression, anxiety, stress, chronic pain, and fatigue, and stress has been shown to worsen MS course.

Meditation has been investigated as a possible beneficial intervention for stress and symptom relief for people with MS. The current literature suggests a beneficial effect of meditation in MS, particularly QOL; however, further research is needed to better understand the potential for meditation as an adjunct to the management of MS.

## Figures and Tables

**Table 1:** Summary of primary literature: studies on meditation/mindfulness in MS.

Author, year, country	Aim	Study type, intervention (if appropriate)	Participant recruitment	Data collected/tools used	Findings	Limitations/comments
Grossman et al. [29] 2010, Switzerland	To examine the effects of a mindfulness-based intervention (MBI) compared to usual care upon quality of life, depression, and fatigue among people with MS.	Randomised controlled trial. Intake interview, 8 weekly 2.5- hour classes, one 7- hour retreat, 40 minutes of homework daily versus usual care (UC) (including medical examination before intervention and six months after intervention).	150 participants with relapsing-remitting or secondary progressive MS recruited from outpatient neurology clinic, other physicians, or advertisements in the Swiss Multiple Sclerosis Society Bulletin. Blinded, randomised group allocation.	Outcomes measured before intervention, after intervention, and at six-month follow-up: Profile of Health-Related Quality of Life in Chronic Disorders (PQOLC), Hamburg Quality of Life Questionnaire in Multiple Sclerosis (HAQUAMS), Center for Epidemiologic Studies Depression Scale (CES-D), Modified Fatigue Impact Scale (MFIS), Spielberger Trait Anxiety Inventory (STAI), Neurologist-administered Neuropsychological assessment, Researcher-devised assessment of personal goal attainment after MBI, and self-reported homework adherence.	Compared to baseline, at postintervention MBI participants showed significant improvements in PQOLC, HAQUAMS, CES-D, MFIS, and STAI greater than the UC group. The benefits remained at six-month follow-up although the effect was lessened for PQOLC and depressive symptoms. The outcomes were not related to gender, EDSS, or taking disease modifying drugs.	Control group not offered sham intervention. Exclusion criteria: other types of MS, Expanded Disability Status Scale score >6 or >1 step increase in previous 12 months, current MS exacerbation, symptomatic medication alteration in previous 3 months, serious psychological disorder or dementia, life threatening or severely disabling physical condition, and others. 5% attrition in intervention group and high attendance rate.

Mills & Allen [32], 2000, United Kingdom	To examine whether mindfulness of movement affects balance and change in symptoms (pilot study).	Randomised controlled trial. Six individual sessions of one-to-one instruction and resources for guidance at home vs. usual care.	12 intervention participants and 12 control participants recruited through local physiotherapists and general practitioners.	Outcomes measured before intervention, after intervention, and at three-month follow-up: Activities of Daily Living questionnaire, test of balance-timed single leg standing, symptom rating questionnaire (21 listed symptoms), and a close relative or friend was also asked to independently assess the degree of change.	The intervention group showed greater likelihood of improvement and less deterioration in symptoms. Measures of overall symptom change showed significant differences between intervention and control groups after intervention and at 3 months. The intervention group also showed improvements in balance which were maintained at 3 months.	All patients with secondary progressive MS and inclusion criteria were having at least one symptom which affected their life on an ongoing basis. Very small sample size 3 dropped out of intervention group and 2 did not return all surveys; 4 did not return all surveys in control group.

Hadgkiss et al. [33] 2013, Australia	To measure change in health-related quality of life one and five years after attending a retreat for people with MS.	Pre- and postintervention (longitudinal follow-up); survey five-day live-in educational retreat promoting lifestyle modification: meditation, healthy diet, vitamin D, and exercise.	274 baseline participants; 196 one-year participants; 96 five-year participants. All enrolled program participants invited to the study.	Outcome measured before intervention and at 1 year and 5 years after intervention: Multiple Sclerosis Quality of Life questionnaire (MSQOL-54)	Significant improvements in physical and mental composite scores and overall quality of life one and five years after attending the retreat.	No control group. Meditation one of several program recommendations. Participants overlap with study by Li et al. [34]

Tavee et al. [45] 2011, United States of America	To determine whether meditation affects pain and quality of life in people with MS and peripheral neuropathy (PN).	Nonrandomised controlled trial. 4-hour introductory session, 8 weekly 90-minute classes on Samatha (breathing), moving and walking meditation and daily practice encouraged versus usual care (UC).	22 intervention participants (10 with MS) and 18 control participants (7 with MS). Recruited patients of a neurological clinic.	Outcomes measured before intervention and after intervention (or baseline and 2 months after UC for controls): 36 item Short Form Health Status Survey (SF-36), Visual Analogue Scale for pain, Patient Determine Disease Steps (measure of disability), 5-item Modified Fatigue Impact Scale (MFIS-5).	After 8 weeks, meditation participants had significant improvements in pain scale, physical, and mental health composite scores and three domains-vitality, physical role, and bodily pain (MS only). Significant improvements in cognitive and psychosocial components of the MFIS for MS meditation group. No change in disability scores for MS meditation group.	Nonrandomised intervention groups assigned based on preference to participate in meditation. Small sample size. High attrition rate. Analysis does not differentiate between MS and PN patients for SF-36. No long term follow-up. Control group not offered sham intervention.

Li et al. [34] 2010, Australia	To measure change in health-related quality of life one and 2.5 years after attending a retreat for people with MS.	Pre- and postintervention (longitudinal follow-up); survey five-day live-in educational retreat promoting lifestyle modification: meditation, healthy diet, vitamin D, and exercise	109 baseline participants; 65 one-year participants; 33 2.5-year participants. All enrolled program participants invited to the study.	Outcome measured before intervention and at 1 year and 2.5 years postintervention: Multiple sclerosis quality of life questionnaire (MSQOL-54).	Significant improvements in physical and mental composite scores and overall quality of life one and 2.5 years after attending the retreat.	No control group. Meditation one of several program recommendations. Participants overlap with study by Hadgkiss et al. [33].

Pritchard et al. [36] 2010, United States of America	To determine whether the practice of Yoga Nidra meditation impacts stress levels for people with MS or cancer.	Pre- and postintervention 6 weekly 90-minute classes and daily practice encouraged.	22 intervention participants (12 with MS). Recruited members of an MS Society.	Outcome measured before intervention and after intervention: Perceived Stress Scale (PSS).	After the completion of the 6-week program, participants had significantly lower PSS scores.	No control group. Small sample size, attrition rate not stated. No demographic or clinical characteristics recorded. Results were shown separately for MS and cancer patients. No long term follow-up.

Senders et al. [42] 2014, United States of America	To evaluate the association between mindfulness, perceived stress, coping strategies, and resilience.	Cross-sectional survey.	119 participants recruited from an outpatient clinic.	Demographics. Clinical characteristics. Perceived Stress Scale. Five Facet Mindfulness Questionnaire Connor-Davidson Resilience Scale. Brief Coping Orientation for Problem Experiences. Social Readjustment Rating Scale. Medical Outcome Study Short Form-36.	After controlling for age, gender, education, disease modifying therapy, type of MS, stressful life events, and disability, trait mindfulness was significantly associated with decreased perceived stress (model accounted for 25% variance), increased resilience (44%), increased adaptive coping (11%), decreased maladaptive coping (29%), and higher mental health QOL (20%).	Mainly recruited from single center. Actual participation in formal mindfulness practice not measured.

Skovgaard et al. [39] 2013, Denmark	To assess and compare characteristics of complementary and alternative medicine (CAM) users and CAM nonusers, and their respective use of CAM and conventional treatments.	Cross-sectional, online survey.	1865 participants recruited from Danish MS society register.	Demographics. Clinical characteristics. Researcher devised and piloted items on CAM use (list of modalities and free text option).	Of the study sample, 91 (4.9%) reporting meditating in the last 12 months.	Self-selecting sample. Recall bias.

Esmonde and Long [41] 2008, United Kingdom	To collect data on the use and benefits of CAM for MS.	Mixed methods, survey and focus group discussions.	138 participants in survey and up to 35 participated in the focus groups. Survey participants recruited from attendees of a national congress of the MS Trust and focus group participants recruited from a workshop at the congress.	Demographics. Clinical characteristics. Researcher devised and piloted questions on use of (last 12 months) and perceived helpfulness of a list of CAM therapies.	34/138 (24.6%) reported using relaxation and meditation. Nearly 40% of those who use relaxation and meditation rate it as “extremely useful,” the remainder rate it as “helpful” or “somewhat helpful.” Benefits of relaxation and meditation described as helping sleep, helping control of spasticity, easing muscle tension, relaxing the mind, clearing the mind, helping control frustration, and sense of well-being (open-ended responses).	Relaxation and meditation not distinguished from each other.

Simmons et al. [40] 2004, Australia	To explore patient views on factors that affect disease onset and progression.	Cross-sectional, online survey.	2529 participants recruited online from a national MS society and the MS International Federation websites.	Demographics. Clinical characteristics. Researcher-devised items on medication and alternative therapy use, diet, and environmental influences.	218/2529 (9%) of participants reported that meditation “improved” their MS; 6/2529 (0.2%) reported that meditation “worsened” their MS. The remaining participants had no view on the effect of meditation on MS.	Unable to verify diagnosis of MS online. Self-selecting sample.

Nayak et al. [37] 2003, United States of America	To explore the use of CAM among a national sample of people with MS.	Cross-sectional survey.	3140 participants recruited from a national mailing list of the MS Foundation.	Demographics. Researcher-devised items on the use of conventional medicines and therapies, and a range of questions about lifetime CAM use including type/frequency/duration/reason/ perceived effectiveness. A checklist of popular CAM therapies was provided.	12.6% of participants reported ever practicing meditation. On a scale from 0–5, the mean (standard deviation) efficacy was 2.06 (1.78) and length of use was 6.10 (7.67) years. The top three symptoms treated by meditation were reported as pain (40.9%), overall symptoms (14.0%), and fatigue (13.4%).	Very large national sample.

Berkman et al. [38] 1999, United States of America	To explore the prevalence of the use of CAM therapies, perceived benefits, harms, and reasons for use.	Cross-sectional survey.	240 participants recruited from a randomly generated list of 500 members of two chapters of the National MS Society (1000 people contacted).	Demographics. Clinical characteristics. Researcher-devised survey on previous and current use of a list of traditional and alternative therapies, rating of how helpful/harmful the therapy is perceived to be, and reason for using therapy (slow progression/symptom relief/emotional relief).	22.9% of the sample had ever used meditation (that is, previous or current use). Benefits of meditation were described as stress relief, relaxation, improved focus, more centered, emotional relief, less fatigue, more positive attitude, strengthened immune system, and slow progression (open-ended responses).	Analysis did not differentiate between meditation and other types of traditional and alternative therapies.
